# The Effects of Social Distance and Front-of-Package Claims on Healthy Food Selection: Moderating Role of Perceived Importance of Eating Healthily

**DOI:** 10.3390/nu15153427

**Published:** 2023-08-02

**Authors:** Veena Chattaraman, Yee Ming Lee, Ebony Marchelle Robinson, Adam J. Book, Fnu Al-Amin

**Affiliations:** 1 Department of Consumer and Design Sciences, Auburn University, Auburn, AL 36849, USA; ajb0183@auburn.edu (A.J.B.); fza0058@auburn.edu (F.A.-A.); 2 Horst Schulze School of Hospitality, Auburn University, Auburn, AL 36849, USA; yzl0085@auburn.edu; 3 Department of Human Development and Consumer Sciences, University of Houston, Houston, TX 77204, USA; emrobin2@central.uh.edu

**Keywords:** construal level theory, front-of-package claims, perceived importance of eating healthily, low-mid SES

## Abstract

Applying construal level theory, this study examined how social distance (thinking of self/children), front-of-package (FOP) claim type (nutrient/health/control), and perceived importance of eating healthily (low/high) impact consumer responses (attitudes/purchase intent) to healthier food products through an online experiment with 171 U.S. parents from low-to-mid socio-economic households. Participants were randomly assigned to view controlled images of healthier foods with packaging that bore different claim types for real and fictitious brands. Results revealed that when choosing for themselves, consumer attitudes were more positive when the healthier food package carried a nutrient (vs. health) claim, however, control claims received the most positive evaluations. When choosing for children, attitudes were more positive when the package carried a health (vs. nutrient/control) claim. Attitudes toward healthier foods were higher for consumers with high (vs. low) perceived importance of eating healthily when the package bore a nutrient claim, however, their attitudes did not significantly differ when the package bore a health/control claim. Purchase intent for healthier foods was higher for consumers with high (vs. low) perceived importance of healthy eating when shopping for self; whereas, when shopping for children, purchase intent did not significantly differ between consumers who varied in perceived importance of eating healthily.

## 1. Introduction

In the United States, obesity remains a common, but preventable, disease [1], impacting two of five adults (42%) [1,2] and one of five children and adolescents aged two through 19 (19%) [2]. Furthermore, obesity is associated with many chronic diseases, including diabetes, heart disease, stroke, and certain cancers [2,3], which are all preventable yet among the leading causes of death and disability in the U.S. [1,2]. Poor dietary habits are among many factors contributing to these phenomena [4]. Hence, remedial action encouraging healthy dietary patterns is critical for preventing obesity and reducing the risk of obesity-related chronic diseases, especially among Americans of low socioeconomic status (low SES), who are shown to have disproportionally higher obesity prevalence (39% vs. 31.2%) [5] and chronic disease mortality rates [6], exasperated by diets high in fat [7], refined grains [7,8], and added sugar [7].

To reduce the prevalence of chronic diseases, public health authorities have implemented preventive strategies focused on nutrition such as the presence of mandatory and voluntary nutritional information on packaged foods. This information is presented either numerically (i.e., Nutrition Facts Panels [NFPs]) and/or non-numerically (i.e., nutrient-content and/or health claims which serve as verbal descriptors of a product). According to the Nutrition Labeling and Education Act (NLEA), an NFP that presents serving size, calories, and other nutrients of the food item is mandatory on packaged foods [9]. Consumers who paid attention to NFPs showed lower intake of fats, saturated fats, cholesterol, and sugars [10] and were more likely to select healthier foods [11,12,13]. However, certain demographic groups that are disadvantaged in terms of income, literacy, and ethnicity have difficulties in understanding NFPs [14,15], thus leading to an increased reliance on FOP claims for evaluating the healthfulness of foods. The third pillar in the 2022 National Strategy on Hunger, Nutrition, and Health is to “empower consumers to make and have access to healthy food choices” ([16], p. 4) facilitated by a “standardized FOP labeling system for food packages to help consumers, particular those lower nutrition literacy, quickly and easily identify foods that are part of a healthy eating pattern” ([16], p. 22). Possible new FOP labeling systems include traffic-light food rating systems, healthy choice checkmark, and star ratings; such systems have been shown to improve healthy food selection among consumers [17,18]. However, before this new standardized system can be identified and mandated on food packages, there is an opportunity to leverage the current FOP labels to prompt healthier food choices among low-income consumers. 

Current FOP labels carry various nutrition claims that imply the healthfulness of a food product [19,20]. These non-numerical claims can be further categorized into health and nutrient claims. Health claims describe the relationship between a food and a certain health condition or disease (e.g., “reduces the risk of heart disease”) while nutrient claims emphasize whether the food is high or low in certain nutrients (e.g., “low in fat”, “good source of fiber”). Some consumers who used non-numerical information on FOP claims were able to identify healthful products [21]. Other consumers found that these claims could be misleading, giving a false sense of security on the nutritional quality of the food products [22,23], a phenomenon that is now labeled as health-washing. Theory-based research is limited regarding which of these claim types (nutrient or health) is effective in promoting healthy food choices; and whether certain individual differences (e.g., perceived importance of eating healthily) increase/decrease the effectiveness of these claims. Perception and beliefs of healthy eating differ between consumers of low versus high income [24] and the efficacy of FOP claim types on healthier food choices among low-income consumers also remains under-researched. The current study aims to address these gaps in knowledge of claim-type effectiveness by investigating whether nutrient claims or health claims (FOP claim type) are more effective in promoting healthier food choices among low-mid SES consumers within decision contexts that prompt thinking about self or children (social distance). We also aim to investigate whether consumers who place low (vs. high) importance of eating healthily respond more positively to:

(a) Nutrient or health claims (FOP claim type) that promote healthier foods; and
(b) Decision contexts that prompt thinking about self or children (social distance) when choosing healthier foods.

## 2. Conceptual Framework and Hypothesis Development

### 2.1. Contrual Level Theory and FOP Claim Type

Construal level theory (CLT), which posits a systematic influence of psychological distance (any type of separation from personal experience) on consumer evaluation and choice [25,26], served as a valuable theoretical framework for the current study. CLT has been applied in a variety of research areas such as morality [26], self-control [27], health promotion [28], and sustainable consumption [29]. In context to the current study, CLT explains consumer response to FOP food claims when choosing healthy food products for self versus children/family. In essence, CLT postulates that consumer response is influenced by various types of psychological distance, including social distance, which is defined as the degree of perceived separation between self and others (e.g., thinking of oneself vs. one’s children/family vs. a stranger) [25]. Further, two basic assumptions underlie CLT: (1) As a stimulus becomes more distant or distal, individuals adopt more abstract construals (i.e., high-level, structured, and holistic representations that focus on the gist of information concerning a stimulus), whereas concrete construals (i.e., low-level, unstructured, and contextualized representations focusing on subsidiary features of a stimulus) are adopted when a stimulus is close or proximal; and (2) The construals (abstract vs. concrete) result in different consumer response [25]. Thus, in the context of food-related decision making, concrete construals address questions such as the number of calories contained in the food and the percentage of added sugars, in other words, nutrition-related information and nutrient claims; whereas abstract construals address questions such as whether the food can prevent heart disease or help with weight management, i.e., health-related information and health claims. 

Previous studies provide support for CLT postulates by linking higher-level (abstract) construals with distal thinking, and lower-level (concrete) construals with proximal thinking [30,31]. These findings imply that, in the context of social distance, when choosing food alternatives for self (proximal thinking), consumers are more likely to place greater importance on the food’s nutrient claim (vs. health claim), whereas when choosing for children or family (distal thinking), the food’s health claim (vs. nutrient claim) is likely to be more important in decisions. Supporting this proposition based on CLT, findings from one study on consumers’ abstract to concrete representations of healthy eating suggest that messages that focus on healthy eating should be framed according to the target consumers’ representation level [i.e., high-level (abstract) messages for distal food choices and low-level (concrete) messages for proximal food choices] [28]. Their study further elucidates that healthy eating is not understood identically by all individuals and holds different levels of value for consumers. In another study investigating how Hispanic participants make healthful food choices based on self-framed (benefits to self) or social-framed (benefits to others) healthy eating messages, Garcia-Collart et al. [32] found that self-framed messages resulted in healthier food selection, suggesting future health marketing messages should target the individual consumer, rather than groups. In a study examining the effect of message tailoring (vs. generic, non-tailored messaging) among low-income households, Clark et al. [33] tailored messages based on CLT by prompting consumers to think proximally about healthy eating. The findings suggested that tailored messages resulted in more favorable outcomes compared to non-tailored messaging. These findings in the context of social distance and food choice directly support the propositions with respect to social distance of the choice context (thinking of self vs. children/family), which has not been examined in previous studies related to food choice and healthy eating. Thus, the following hypotheses are proposed:

**H1a.**  Proximal thinking (choosing food products for self) will exhibit greater response (attitudes/purchase intent toward healthier foods) to nutrient claims than health claims on food packaging.

**H1b.**  Distal thinking (choosing food products for children) will exhibit greater response (attitudes/purchase intent toward healthier foods) to health claims than nutrient claims on food packaging.

### 2.2. Moderating Role of Perceived Importance of Eating Healthily

Healthy eating beliefs have been found to lead to healthy behaviors [32,34]. In the current study, healthy eating belief was conceptualized through the perceived importance of eating healthily. The elaboration likelihood model (ELM), another theory of decision-making that has been widely applied in consumer and health research [35,36,37], places consumers on a quantitative elaboration continuum between effortful and heuristic processing, wherein the amount and nature of object-relevant elaboration is determined by individual and situational factors [38]. Specifically, as motivation and/or ability to evaluate an object decreases, people rely on heuristic processing in forming their evaluations, whereas as motivation and/or ability increases, people employ effortful and object-relevant information processing [38]. Consumers who place higher importance on eating healthily have been found to use more effortful processing of nutritional information of packaged foods [39]. With respect to health claims, previous literature showed that motivation to process is a predictor of health-claim use, and this motivation was strongly related to their interest in healthy eating [40]. In another study investigating how differences in nutrition knowledge (experts vs. novices) influenced women’s interpretations of intrinsic (e.g., ingredient list) and extrinsic cues (e.g., health and nutrient claims) on food labels, the researchers found that experts employed effortful processing by focusing on intrinsic cues, whereas novices used heuristic processing by focusing on extrinsic cues [41]. Results from the two studies mentioned above [40,41] suggest that consumers with high (vs. low) perceived importance of eating healthily are more likely to process/elaborate on health or nutrient claims. However, prior research has not uncovered which of these two claim types (nutrient vs. health) are more effective for these consumer groups (high vs. low perceptions of healthy eating importance). Hence, there is opportunity to further regulate and optimize health and nutrient claims to benefit healthy food selection for such consumers. A study on consumers’ sustainable choices found that psychological distance effects have lower influence on high elaboration consumers since they often navigate both proximal and distal thinking in reaching decisions, whereas low elaborators are more influenced by proximal, concrete thinking when making product decisions [42]. Sustainable decision-making makes distal choices more salient. However, that need not be true in the case of food choices, emphasizing the need to examine whether consumers who place low versus high importance on eating healthily respond to healthier foods differentially, when thinking about themselves (proximal) or their children (distal). Given the lack of research on this topic, we present the following research question:

*RQ 1: Does perceived importance of eating healthily (low vs. high) moderate the effects of (a) FOP food claim type (nutrient/health/control), and (b) social distance (thinking of self vs. children) on consumer response (attitudes and purchase intent) toward healthier foods?*

## 3. Materials and Methods

### 3.1. Experimental Design

The current study employed an online quasi-experiment consisting of a 2 (social distance: thinking of self vs. thinking of children) × 3 (FOP claim type: nutrient, health, or control) × 2 (perceive importance of eating healthily: low vs. high) mixed factorial design. Social distance and FOP claim type were the manipulated independent variables and between-subjects factors. Healthy product category (whole grain wheat and low-fat dairy) and brand name (real and fictitious) were within-subjects factors. The use of two product categories and real/fictitious brands as within-subjects factors allowed for controlling product- and brand-specific preferences in the experiment. The perceived importance of eating healthily (low vs. high) was a measured independent variable, assessed with a scale adopted from Chandon and Wansink’s study [39]. Consumers attitudes and purchase intentions were measured as the dependent variables.

### 3.2. Sample

Given the relevance of SES to health outcomes [5,43,44], this study targeted low-mid SES consumers who make food purchasing decisions for their households. A total of 175 consumers aged 19–64 with at least one child (under the age of 19) living with them were recruited from Prolific (https://www.prolific.co), a nationwide online panel, based on the MacArthur Scale of Subjective Social Status, a subjective indicator of SES [45]. Specifically, consumers who self-identified as placing within levels 1–5 (with 10 being the highest) on the socioeconomic status ladder were included in the study. Four respondents who failed the attention check questions were omitted from the sample. Thus, the usable sample size was 171 (see Table 1), consisting of more female consumers (66.1%). The respondents’ ages ranged from 20 to 61 years, with a mean age of 33.7 years (*SD* = 7.01). As depicted in Table 2, most of the respondents were non-Hispanic White (76%), followed by non-Hispanic Black (10.5%), Hispanic (8.2%), Asian (2.9%), and other (2.3%). 

### 3.3. Stimuli, Procedure, and Measures

Participants meeting the research criteria were randomly assigned to one of six conditions in Table 3. The stimuli included a series of healthier options of wheat products (whole grain) and dairy products (low fat) from real and fictitious brands (to control for brand familiarity effects) containing the claim variations that aligned with their assigned experimental condition (see examples in Table 3 and Appendix A for the complete set of stimuli). The stimuli consisted of digital prototypes of the product packages created and/or manipulated using Affinity Designer and Affinity Photo software (versions 1.10.6). For the real brands, images of product packages (available online) were manipulated to reflect the different claim conditions. Products that are commonly consumed by consumers in low-mid income ranges were selected for the study (cereal, pasta, milk, and yogurt). To induce social distance in their thinking, participants read a scenario that instructed them to imagine shopping for themselves (participants) or their children and then evaluate a target product with a health/nutrient/control claim. The target products varied for self- and children-scenarios such that self-scenario participants evaluated whole grain pasta and low-fat yogurt for real and fictious brands, whereas children-scenario participants evaluated whole grain cereal and low-fat milk for real and fictitious brands. Hence, each participant saw four product stimuli (two wheat and two dairy products) presented in random order to control for order effects. Health claims were consistent with FDA-approved claims that state the relationship between a food and the risk reduction of a disease [46]; the wording of the nutrient claims was formulated in accordance with the FDA’s regulations [47]. For whole grain wheat products, health claims emphasized reduced risk of heart disease, and nutrient claims promoted dietary fiber, B vitamins, and minerals. For low-fat dairy products, health claims emphasized reduced risk of osteoporosis, and nutrient claims promoted protein, vitamin D, and calcium. The control condition either included no claim or a claim with no nutritional information. Following each target product presentation, participants completed measures for attitudes and purchase intentions (DVs). Following evaluation of all four target products, participants completed the manipulation checks, a scale measuring perceived importance of eating healthily, and demographic items (see Appendix B for a list of all measurements).

## 4. Results

The data were analyzed using the Statistical Package for the Social Sciences (SPSS version 27.0). Data analysis consisted of (a) preliminary analyses (data cleaning, sample profiling, validity, and reliability checks) employing descriptive statistics and factor analysis, and (b) manipulation checks and hypothesis tests using analysis of variance (ANOVA).

### 4.1. Preliminary Analysis

Prior to creating composites for all variables, reliability and unidimensionality was assessed for each scale. Reliability analysis using Cronbach’s α suggested adequate reliability, as α was greater than 0.88 for all scales. An exploratory factor analysis (EFA) using principal component analysis with Varimax rotation indicated that each variable comprised only one factor. The item scores from each scale were then averaged to produce a composite variable score for hypotheses testing. Finally, participants were split into two groups (low and high) based on the median scores for perceived importance of eating healthily. Those with scores below the median score (5.20) were coded as having low perceived importance of eating healthily (*n* = 94, *M* = 4.25, *SD* = 0.99), while participants with scores above the median were coded as having high perceived importance of eating healthily (*n* = 77, *M* = 6.15, *SD* = 0.50). 

### 4.2. Manipulation Checks

A manipulation check is a statistical test that is conducted to evaluate the effectiveness of an independent variable manipulation in an experimental design [48]. As discussed previously, the independent variables, social distance, and FOP claim type were manipulated through shopping scenarios and the claim content of the FOP labels (see Table 3), respectively. The results from a series of one-way ANOVAs indicated that the social distance and FOP claim type manipulations were successful. Specifically, the success of the scenario that participants were provided to ‘shift’ their thoughts towards themselves (proximal condition) or their children (distal condition) was determined through one-way ANOVA results, which revealed that participants in the distal condition were significantly more likely to think of their children when shopping (*M* = 6.37, *SE* = 0.09; *F*_1,169_ = 59.42, *p* < 0.001) compared to those in the proximal condition (*M* = 4.60, *SE* = 0.21). Similarly, participants in the proximal condition were significantly more likely to think of themselves when shopping (*M* = 5.48, *SE* = 0.16; *F*_1,169_ = 16.83, *p* < 0.001) compared to those in the distal condition (*M* = 4.49, *SE* = 0.19). With respect to the manipulation check for FOP claim type, a one-way ANOVA revealed that participants who were presented with packaging that bore a health claim were significantly more likely to believe that the packaging contained a claim that the product reduced risks of certain diseases (*M* = 5.94, *SE* = 0.18; *F*_2,168_ = 43.35, *p* < 0.001) compared with those presented with a nutrient claim (*M* = 3.38, *SE* = 0.24) or control claim (*M* = 3.27, *SE* = 0.24). Participants who saw packaging that bore a nutrient claim were more likely to believe that the packaging contained a claim that the product was a good source of nutrients and/or vitamins (*M* = 5.93, *SE* = 0.13; *F*_2,168_ = 8.65, *p* < 0.001) compared with those who saw a health claim (*M* = 5.27, *SE* = 0.22) or a control claim (*M* = 4.91, *SE* = 0.18).

### 4.3. Hypotheses Testing

The hypotheses and research questions were tested through two, three-way repeated measures ANOVAs with FOP claim type, social distance, and perceived importance of eating healthily as between-subjects factors, product category (wheat and dairy) and brand type (real and fictitious) as within-subjects factors, and consumer attitudes and purchase intentions as dependent variables for each analysis. The results of the between-subjects effects revealed a significant interaction effect of FOP claim type and social distance on consumer attitudes (*F*_2,159_ = 3.80, *p* = 0.024, *η*^2^ = 0.05) but not purchase intentions (*F*_2,159_ = 0.489, *p* = 0.614). Apart from this hypothesized interaction effect, there were no significant main effects of FOP claim type on consumer attitudes (*F*_2,159_ = 1.71, *p* = 0.185) or purchase intentions (*F*_2,159_ = 2.40, *p* = 0.094). There were also no significant main effects of social distance on consumer attitudes (*F*_1,159_ = 0.01, *p* = 0.928) or purchase intentions (*F*_1,159_ = 0.35, *p* = 0.556). The following paragraphs report the results for each hypothesis and research question.

**Figure 1:**
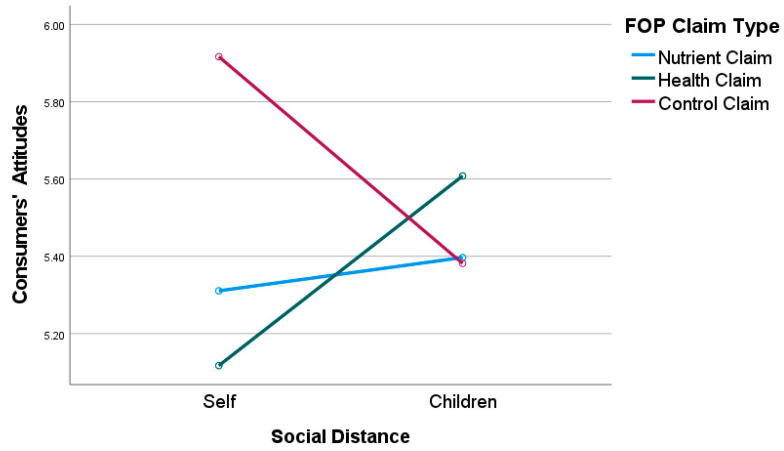
Interaction effect of FOP Claim Type × Social Distance on Consumer Attitudes.

Hypothesis 1a: The ANOVA results for the significant interaction effect of FOP claim type and social distance on consumer attitudes as the dependent variable revealed that consumers who engaged in proximal thinking (i.e., choosing products for themselves) exhibited more positive attitudes toward healthier foods with nutrient claims than health claims on food packaging, as predicted in H1a; however, consumer attitudes were most positive when the healthier foods contained the control claim (*M_control_* = 5.92, *SE* = 0.18, *M_nutrient_* = 5.28, *SE* = 0.20, *M_health_* = 5.12) [see Figure 1]. In context to purchase intentions, since the interaction effect for FOP claim type and social distance was not significant (*F*_2,159_ = 0.489, *p* = 0.614), the simple effects are not interpreted. Hence, H1a was supported partially in context to consumer attitudes but not purchase intentions.

Hypothesis 1b: Consistent with predictions in H1b, the ANOVA results for the significant interaction effect of FOP claim type and social distance on consumer attitudes, as the dependent variable, revealed that consumers who engaged in distal thinking (i.e., choosing products for their children) demonstrated more positive attitudes toward healthier foods that bore health claims on food packaging compared to nutrient claims or control claims (*M_health_* = 5.58, *SE* = 0.22, *M_nutritient_* = 5.39, *SE* = 0.19, *M_control_* = 5.39) [see Figure 1]. Regarding purchase intentions, the interaction effect of FOP claim type and social distance was not significant, hence the simple effects are not interpreted. Thus, H1b is only partially supported for consumer attitudes but not purchase intentions.

**Figure 2:**
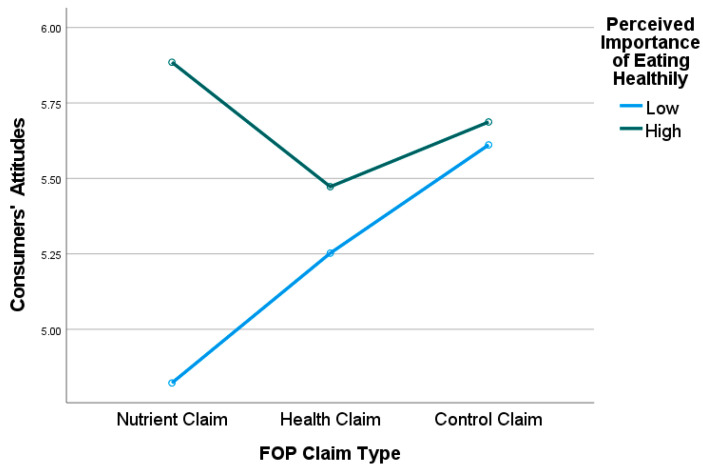
Interaction effect of FOP Claim Type × Perceived Importance of Eating Healthily on Consumers’ Attitudes.

Research Question 1a: A research question was posed on whether consumer-perceived importance of eating healthily moderates the main effect of FOP claim types on consumers’ attitudes and purchase intentions toward healthier foods. The results of the between-subjects effects revealed a significant two-way interaction effect for FOP claim type and perceived importance of eating healthily on consumers’ attitudes (*F*_2,159_ = 4.14, *p* = 0.018, *η*^2^ = 0.05) but not purchase intentions (*F*_2,159_ = 1.62, *p* = 0.200). Perceived importance of eating healthily had a significant effect on consumer attitudes for foods containing nutrient claims (*F*_1,159_ = 16.13, *p* < 0.001, *n*^2^ = 0.09) but not foods containing health claims (*F*_1,159_ = 0.58, *p* = 0.447) or control claims (*F*_1,159_ = 0.09, *p* = 0.760). Specifically, pairwise comparisons demonstrated that when evaluating healthier products with a nutrient claim, consumers with high perceived importance of eating healthily had significantly more positive attitudes compared to those with low perceived importance of eating healthily (*M_high_* = 5.89, *SE* = 0.19, *M_low_* = 4.82, *SE* = 0.18) [see Figure 2]. There was no significant difference between those with high versus low perceived importance of eating healthily when evaluating healthier products with health claims (*M_high_* = 5.47, *SE* = 0.23, *M_low_* = 5.25, *SE* = 0.18) or control claims (*M_high_* = 5.69, *SE* = 0.18, *M_low_* = 5.61, *SE* = 0.17) [see Figure 2]. 

**Figure 3:**
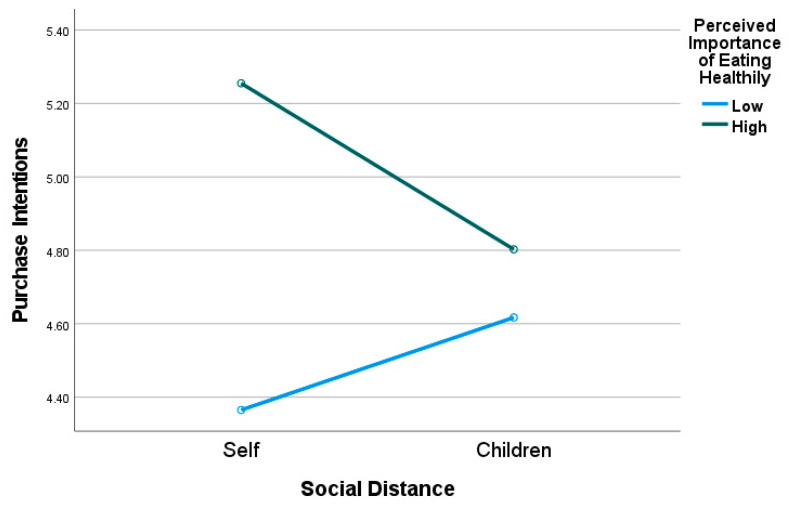
Interaction effect of Social Distance × Perceived Importance of Eating Healthily on Consumers’ Purchase Intentions.

Research Question 1b: Similarly, a research question was posed on whether consumers’ perceived importance of eating healthily moderates the main effect of social distance on consumer response (i.e., consumers’ attitudes and purchase intentions) toward healthier foods. The results of the between-subjects effects revealed a significant two-way interaction effect for social distance and perceived importance of eating healthily on consumers’ purchase intentions (*F*_1,159_ = 4.30, *p* = 0.040, *η*^2^ = 0.03) but not attitudes (*F*_1,159_ = 1.36, *p* = 0.245). Perceived importance of eating healthily had a significant effect for consumers engaged in proximal thinking (i.e., choosing products for themselves) (*F*_1,159_ = 14.04, *p* < 0.001, *n*^2^ = 0.08) but not for those engaged in distal thinking (*F*_1,159_ = 0.59, *p* = 0.445). More specifically, as shown in pairwise comparisons, when choosing healthier products for themselves, consumers with high perceived importance of eating healthily had significantly higher purchase intentions for healthier food products compared to those with low perceived importance of eating healthily (*M_high_* = 5.26, *SE* = 0.18, *M_low_* = 4.37, *SE* = 0.16). As demonstrated in Figure 3, there were no significant differences between consumers with high versus low perceived importance of eating healthily when engaged in distal thinking (evaluating products for their children) (*M_high_* = 4.80, *SE* = 0.18, *M_low_* = 4.62, *SE* = 0.16, *p* = 0.445).

## 5. Discussion

In this study, we examined the effects of social distance, FOP claim type, and perceived importance of eating healthily on consumer response to healthy food products. CLT was used as the theoretical framework and the theory states that abstract, high-level (vs. low-level) mental representations are more salient as psychological distance increases and that concrete, low-level (vs. high-level) representations are more salient as psychological distance decreases [25,26]. An online experiment with 171 low-mid SES U.S. consumers offered support for expectations that consumer response to packaging claims is influenced by psychological social distance and perceived importance of eating healthily.

The results of this study support H1a, in that more positive attitudes were observed among participants following exposure to a low-level, nutrient (vs. health) claim on healthier food packaging when participants were choosing for themselves (proximal thinking). Notably, the most favorable responses were observed when the healthier food package bore a control claim (vs. nutrient and health claims). This finding may, in part, be related to skepticism of food advertising claims [49,50]. For instance, Mitra et al. [51] found that consumers were skeptical about health claims because they have been bombarded and jaded by these claims in the marketplace, resulting in disinterest among them. In line with construal level theory [25,26], consumers thinking about their children (distal) revealed more positive attitudes when observing high-level, health claims (vs. nutrient claims) on packaging. According to prior studies, parents place a significantly higher value on their children’s health compared to their own health, amounting to nearly a two-fold difference [52,53]. This may explain why health claims are valued more than nutrient claims on FOP labels in context to food choice for children. No significant effect was revealed for the interaction between FOP claim type and social distance on purchase intentions. Purchase intent is influenced by multidimensional factors beyond message framing such as need for the product, an evaluation of price of the product (not employed in the current study), and brand preference [54,55,56,57]. Hence, these results need not imply that targeting FOP claim types based on social distance will not impact purchase intent, but rather that the above factors may need to be measured and controlled for in order to reveal any significant effects in context to purchase intent. 

Consistent with Gupta et al.’s [19] findings on FOP claims and healthy-consumption attitudes, a significant interaction effect for FOP claim type and perceived importance of eating healthily on consumer attitudes was found. Perceived importance of healthy eating had a significant effect on consumer attitudes for foods containing nutrient claims but not health or control claims. In line with links identified between need and interest for healthy eating [40], consumers who placed high importance on eating healthily had significantly more positive attitudes when evaluating healthier products that bore a nutrient claim. However, no significant difference between those who place high importance on eating healthily versus those who place low importance on eating healthily was observed when healthier products bearing health claims were observed or when there was a control claim. 

Consumers who engaged in proximal (self-related) thinking and placed high importance on eating healthily had significantly higher purchase intentions for healthier food products compared to those who did not place such importance. Consumers who engaged in distal (children-related) thinking did not reveal a significant difference between those who placed high versus low value on healthy eating when evaluating products. In other words, the perceived importance of eating healthily comes into play when making food purchases for the self. This finding is consistent with Garcia-Collart et al. [32], who suggested that future health marketing messages should target the individual consumer rather than groups, based on their results related to self and socially framed messages. Conversely, consumers’ intent to purchase healthy food did not depend on the level of perceived importance of eating healthily when buying for their children. In other words, both groups of consumers (i.e., those who placed high vs. low importance on eating healthily) made equally healthful food selections for their children converging with previous studies that have demonstrated that parents tend to prioritize the health outcomes of their children more than their own and place almost twice as much value on their child’s health as they do on their own [52,53]. This finding importantly suggests that parents’ food choices are normatively geared toward healthier options for children. However, parents’ food choices for self vary depending on health-related inner motivations, such as perceived importance of eating healthily.

## 6. Theoretical and Practical Implications

Theoretically, this study applies CLT to understand consumer decision-making related to healthy food choices. Specifically, this study presents the role of construal level (proximal and distal) in context to social distance in decision-making on FOP claim evaluations of consumers. The findings of this study suggest that consumer attitudes toward food claim types varied, depending on whether food choices were made for consumers themselves (proximal) or their children (distal), providing support for CLT in a highly relevant decision context that has not been examined previously. Support for construal-level-based behavioral change warrants further exploration in other health-related messaging contexts, such as in-store signage and online advertising for healthy foods. Additionally, this study investigated the moderating role of perceived importance of eating healthily, an individual difference variable, providing a sound theoretical integration of message, individual, and contextual (social distance) variables. 

From a practical perspective, the results of this study inform the food industry on messaging strategies based on construal level (self vs. children) for various food categories. The control claim on FOP labels was indeed the most favorable when thinking about self; however, when thinking about children, the health claim led to the most positive responses. Given growth in organic produce distribution [58], opportunity is ripe within organic and health-focused food spaces to engage in tailored messaging based on construal level. Grocers and food brands may realize improved return on marketing spend by planning advertising to align with anticipated consumer social distance. Specifically, the findings of our study suggest that FOP health claims may be more effective for healthy food products such as healthier cereals, lunch snacks, and mac and cheese (healthier vesion) that target children. As health conditions of diabetes, heart disease, and obesity persist in an environment of escalating healthcare costs [59,60,61,62], policymakers are faced with the challenge of guiding healthier food consumption. Public policymakers will be supported by findings that demonstrate messaging strategies that can be leveraged for promoting healthy food consumption. Such strategies can be employed to improve dietary choices parents make for children by promoting health- rather than nutrient-based communication. 

On the other hand, individuals making food choices for themselves responded most favorably when the control claim was presented. Although this study did not measure skepticism and consumers’ level of trust in FOP claims (health and nutrients), previous literature indicated that adult consumers were skeptical about the credibility of the FOP claims [51,63]. They also demonstrated a lower level of trust towards FOP claims and tended to verify these claims with the information presented on NFPs [51,63]. Therefore, it was possible that the participants in this study preferred the control claims when no other way of verification was presented to them. The government should ensure that greater regulations are in place to increase the credibility of claims, thus ensuring higher consumer confidence, and greater utilization of the claims as a source of nutrition information when making food selection and purchase decisions.

Despite its contributions to the literature on FOP claims and CLT, this study has the following research limitations. First, there may be concern with prototype fidelity since the digital renderings of food packages were displayed in a digital format, embedded in the online survey. The variations in screen sizes and qualities of the screens could lead to display inconsistencies, affecting how the participants responded to the questions. Second, this study employed only four food products (cereal, pasta, milk, and yogurt) as the carriers of health and nutrient claims. Other food commodities could also be purchased and consumed by low-income consumers. Future research may benefit from focus groups to identify food products most relevant to low-income consumers prior to stimuli development to better target low-income consumer needs and purchase patterns. Third, participants were recruited through the Prolific panel, an online research platform for recruiting research participants, and the hypotheses have not been tested in a real store setting. Future research can be conducted in a physical store or in a controlled, simulated grocery store environment to further verify the findings of this current study. Fourth, this study measured attitudes and purchase intentions, not actual purchase. Since actual behavior is known to be different from attitudes and behavioral intentions [60], future studies could measure actual purchase, for example, via transactional data to further investigate the effects of various types of FOP claims in different decision contexts. 

## Figures and Tables

**Table 1:** Sample size within experimental conditions.

Variable	Condition	*n*
Social Distance	Thinking of Self	89
	Thinking of Children	82
FOP Claim Type	Nutrient Claim	56
	Health Claim	51
	Control Claim	64

Note. *N* = 171.

**Table 2:** Participants’ characteristics (*N =* 171).

Characteristics	*n*	*%*
**Sex**		
Male	57	33.3
Female	113	66.1
Prefer not to say	1	0.6
**Ethnic Group**		
Asian/Pacific Islander	5	2.9
Hispanic	14	8.2
Non-Hispanic Black	18	10.5
Non-Hispanic White	130	76
Other (Please specify)	4	2.3
**Geographical Region**		
Midwest (IA, IL, IN, KS, MI, MN, MO, ND, NE, OH, SD, WI)	44	25.7
Northeast (CT, DC, DE, MA, MD, ME, NH, NJ, NY, PA, RI, VT)	25	14.6
Southeast (AL, AR, FL, GA, KY, LA, MS, NC, SC, TN, VA, WV)	47	27.5
Southwest (AZ, NM, OK, TX)	21	12.3
West (AK, CA, CO, HI, ID, MT, NV, OR, UT, WA, WY)	34	19.9
**Education**		
Some high school	4	2.3
High school diploma	37	21.6
Some college or technical school	69	40.4
College degree (4 Years)	48	28.1
Some graduate school	4	2.3
Graduate Degree (Master’s, Doctorate, etc.)	9	5.3
**Income**		
$25,000 and below	33	19.3
$25,001–$50,000	73	42.7
$50,001–$75,000	39	22.8
$75,001 and above	26	15.2
**Marital Status**		
Single	36	21.1
Married	80	46.8
Living as married	37	21.6
Separated	9	5.3
Divorced	8	4.7
Widowed	1	0.6
**Health Status**		
Poor	10	5.8
Fair	44	25.7
Good	79	46.2
Very Good	32	18.7
Excellent	6	3.5

**Table 3:** Fictitious brands: whole grain wheat and low-fat dairy products with FOP claim type.

**Thinking of Self + ** **Nutrient Claim**	**Thinking of Self + ** **Health Claim**	**Thinking of Self + ** **Control Claim**
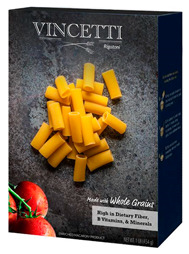	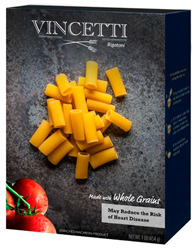	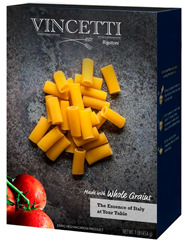
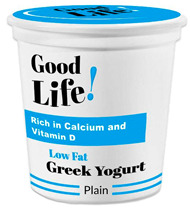	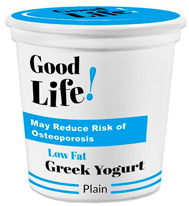	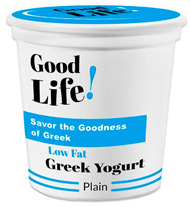
**Thinking of Children + ** **Nutrient Claim**	**Thinking of Children + ** **Health Claim**	**Thinking of Children + ** **Control Claim**
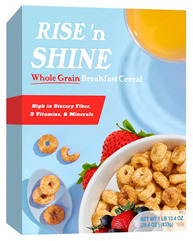	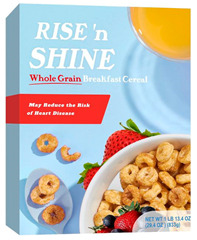	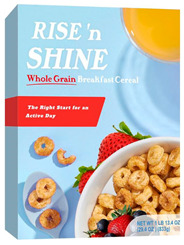
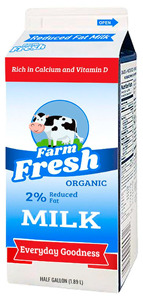	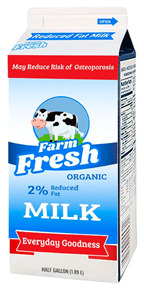	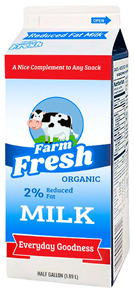

Source: Digital prototypes created by co-author, A.J.B.

## Data Availability

The data supporting the findings of the current study are available from the corresponding author.

## Appendix A

FOP Claim Types for Existing and Fictitious Brands.

	**FOP Claim Type**		
	**Nutrient**	**Health**	**Control**
Whole Grain Wheat Product Stimuli for Real Brands			
Pasta (Self): Barilla			
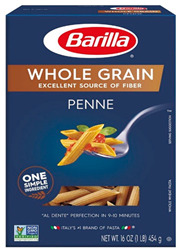
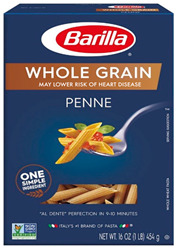
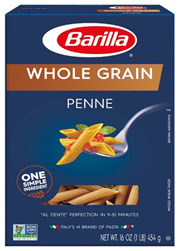
Claim Wording	Excellent Source of Fiber	May Lower Risk of Heart Disease	No Text
Cereal (Children): Honey Nut Cheerios			
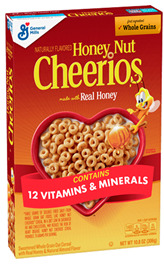
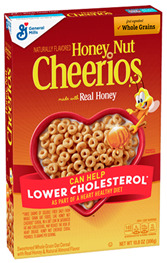
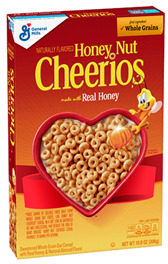
Claim Wording	Contains 12 Vitamins and Minerals	Can Help Lower Cholesterol as Part of a Heart Healthy Diet	No Text
Whole Grain Wheat Product Stimuli for Fictitious Brands			
Pasta: Vincetti (Self)			
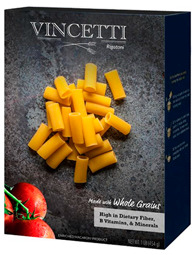
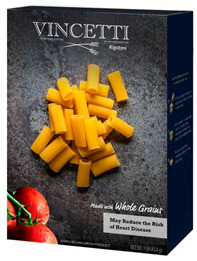
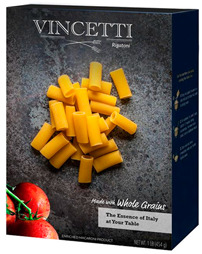
Claim Wording	High in Dietary Fiber, B Vitamins, and Minerals	May Reduce the Risk of Heart Disease	The Essence of Italy at Your Table
Cereal: Rise ‘n Shine (Children)			
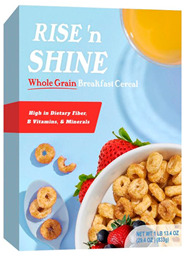
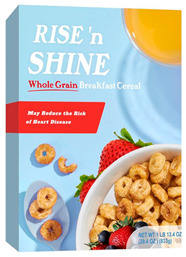
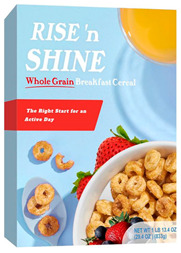
Claim Wording	High in Dietary Fiber, B Vitamins, and Minerals	May Reduce the Risk of Heart Disease	The Right Start for an Active Day
**Low Fat Dairy Product Stimuli for Real Brands**			
Yogurt: Dannon (Self)			
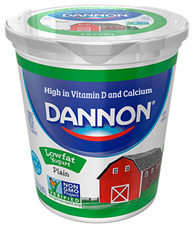
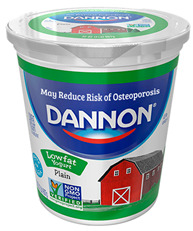
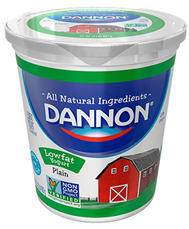
Claim Wording	High in Vitamin D and Calcium	May Reduce Risk of Osteoporosis	No Text
Milk: Organic Valley (Children)			
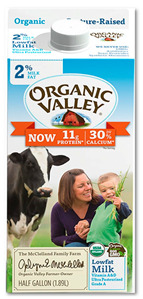
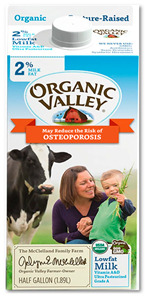
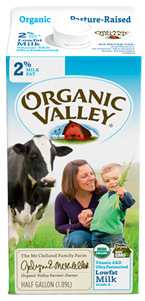
Claim Wording	Now 11 g Protein * 30% DV Calcium * * Per Serving	May Reduce the Risk of Osteoporosis	No Text
Low Fat Dairy Product Stimuli for Fictitious Brands			
Yogurt: Good Life! (Self)			
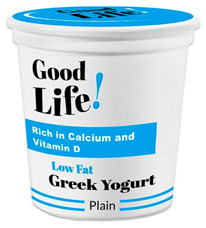
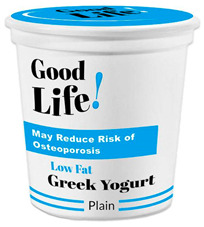
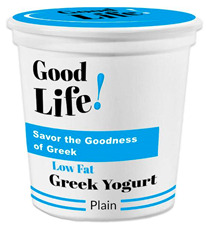
Claim Wording	Rich in Calcium and Vitamin D	May Reduce Risk of Osteoporosis	Savor the Goodness of Greek
Milk: Farm Fresh Organic (Children)			
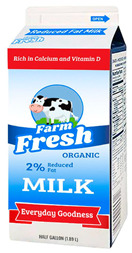
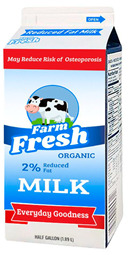
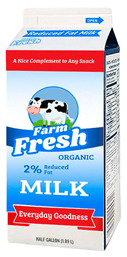
Claim Wording	Rich in Calcium and Vitamin D	May Reduce Risk of Osteoporosis	A Nice Complement to Any Snack

## Appendix B

List of Measurements.

**Variable**	**Items**	**Source**	**α**
Social Distance Manipulation Check	7-point Likert-type scale [1= Strongly Disagree (SD); 7 = Strongly Agree (SA)] • The situations made you think about yourself. • The situations made you think about your child/children.	Garcia-Collart et al., 2020 [32]	
Message Value Manipulation Check	7-point Likert-type scale (1= SD; 7 = SA) • The packaging contained a claim that the product is a good source of nutrients and/or vitamins. • The packaging for the selected products contained a claim that the product may reduce risk of certain diseases.	Authors	
Perceived importance of eating healthily	7-point Likert-type scale (1= SD; 7 = SA) • I watch what I eat. • I pay attention to what I eat. • I pay attention to how much I eat. • Eating healthily is important to me. • I pay attention to nutritional information.	Chandon and Wansink, 2007 [39]	0.91
Purchase Intention	7-point Likert-type scale (1= SD; 7 = SA) • I would consider purchasing this product for myself (my child/children). • I intend to purchase this product for myself (my child/children). • I would recommend this product to others (for their child/children).	Berezowska et al., 2015 [64]	0.90–0.93
Attitudes	7-point semantic differential scale • Bad/Good • Unpleasant/Pleasant • Unfavorable/Favorable	MacKenzie and Lutz, 1989 [65]	0.95–0.97
